# Selective effects of psychosocial stress on plan based movement selection

**DOI:** 10.1038/s41598-022-09360-0

**Published:** 2022-03-30

**Authors:** Sarah E. M. Stoll, Leonie Mack, Jean P. P. Scheib, Jens Pruessner, Jennifer Randerath

**Affiliations:** 1 Department of Psychology, University of Konstanz, 78464 Constance, Germany; 2 Lurija Institute for Rehabilitation and Health Sciences at the University of Konstanz, Schmieder Foundation for Sciences and Research, 78476 Allensbach, Germany

**Keywords:** Psychology, Human behaviour

## Abstract

Efficient movement selection is crucial in everyday activities. Whether this function is governed by our stress system is so far unknown. In the current study, data from thirty-six young male adults were analyzed. They performed rule- and plan-based movement selection tasks before (session 1) and after (session 2) a psychosocial stressor, or after a control condition without additional social stressor. Results showed that the rule-based efficiency advantage which was observed prior to the psychosocial stressor was significantly reduced afterwards in the whole sample, as well as in the stress group. Regression analyses revealed that this effect was due to a modulation of the plan-based approach. Especially variations—both increase and decrease—in the parasympathetic activity (reflected by the heart rate variability measure RMSSD) appeared to be disadvantageous for plan-based movement selection improvement. In contrast, performance in the rule-based movement selection tasks appeared to be rather invariant to external influences. The current results suggest that autonomic nervous system activity might modulate motor-cognitive performance. This modulatory capability might be selective for plan-based approaches, hence the applied strategy to movement selection could be decisive when it comes to the vulnerability of motor-cognitive processes towards psychosocial stress.

## Introduction

As humans we are constantly required to use our sensorimotor system to adequately interact with objects around us in response to implicit and explicit rules, for example hitting the brake to avoid a collision while driving. Humans may use different approaches to action when it comes to movement selection. Two prominent approaches to movement selection are rule- and plan-based approaches.

Rule-based behavior contributes to movement selection as a fixed stimulus–response mapping^1^. This means a specific stimulus is associated with a specific response. For example, red traffic lights (stimulus) will lead the car driver to brake (response). The fixed mapping between stimuli and responses stated in rules can be exploited to support the initiation of a desired behavior. These can be described as implementation intentions^2,3^, which are rules in the form of if–then-sentences (“If a red light appears, then stop.”). Several past studies of our group indicated the potential of if–then rules to serve as a reliable strategy to appropriate movement selection^4–7^.

An alternative strategy for movement selection involves flexible stimulus–response mappings, i.e., a stimulus is associated with multiple responses. Movement selection under such conditions may be solved for example by use of prospective planning. For incidence, the handle of a cabinet door (stimulus) can be grasped differently (e.g., pronated versus supinated grasping response) to open it, depending on the handle’s location (above or below the actor) and the respective subsequent movement (e.g., door opens downwards or upwards). Rosenbaum and colleagues observed that people tended to select the specific initial grip type in such a way (supinated versus pronated) that allows them to finish the movement in a comfortable position, the so-called end-state comfort effect^8–10^. The end-state comfort effect has been replicated in different conditions of object interactions such as, for example, common tool-use^11–15^.

We^5^ have previously introduced an experimental setting that allows to compare rule- and plan-based movement selection approaches, which both lead to similar movements. In the so-called rule-plan-motor-cognition (RPMC) paradigm, healthy young participants used either a previously learned rule or prospective planning to choose between a pronated or a supinated grip. Subsequently, participants perform a rotating movement. The results showed that the rule-based approach is more efficient in terms of a reduced time interval from stimulus presentation to action initiation (i.e., response-time). This finding was replicated and extended in Randerath et al.^6^ by the implementation of the RPMC paradigm in a brain imaging study using fMRI repetition suppression. There we found similar cortical areas to be involved in plan- and rule-based movement selection, but for the rule-based approach the cortical activation was weaker, suggesting an advantage of the rule-based approach in neuro-metabolic demand, too. However, the efficiency discrepancy between rule- and plan-based approaches can be modulated. In a recent study of our group, we^7^ observed that the efficiency advantage for the rule-based approach increased when task difficulty was elevated.

To the best of our knowledge, thus far it has not been investigated how acute social-evaluative stress may affect the performance in humans of such relatively simple grasping movements selected based on planning versus rule retrieval. Motor-cognitive behavior frequently occurs in the presence of a stressful environment, for example when operating a car in dense traffic, observed by possibly impatient other drivers while trying to reach a destination on time. Here, both rule-based fixed stimulus–response mappings (e.g., “if there is a red traffic light then hit the brake, if there is a green traffic light then press gas pedal”) as well as plan-based flexible stimulus–response mappings (e.g., when entering a roundabout: execute the correct motor responses at the appropriate time to indicate directions, operate the steering wheel and shift gears) are needed for efficient maneuvering. Based on above-described findings by Scheib et al.^7^, showing enhancement of the rule-based efficiency advantage under conditions of elevated difficulty and assuming social-evaluative stress to be a straining factor, one hypothesis would be that the advantage of rule-based movement selection (i.e., the rule-based efficiency effect) would be similarly enhanced when being exposed to stress.

Chronic, but also acute stress negatively impacts on functioning, physical and mental well-being and in the long run, the health of the individual^16–18^. Since the early findings by Yerkes and Dodson^19^, it is well known that varying levels of arousal or stress modulate performance depending on the complexity of the given task. Several more recent studies demonstrated that cognitive functioning including the motor system appears to be susceptible to the effects of acute stress. Depending on the specific cognitive construct in question, studies report heterogeneous results. While social-evaluative stress was often reported to have a negative impact on cognitive flexibility^20–22^, mixed findings were reported for working memory^23,24^ and for decision making^25,26^. Inconsistent findings were also reported for memory retrieval^27^, i.e., enhanced^28^ or decreased performance^29^ after stress. For memory functions, Schwabe et al.^30^ proposed an integrative model on the effects of stress, which describes the distinct temporal interplay of physiological and endocrine stress responses (catecholamines and glucocorticoids) on the one hand and memory encoding, consolidation, and retrieval on the other hand. Both the autonomic nervous system through catecholamines and the endocrine stress system through glucocorticoids have central nervous system effects which are believed to be responsible for these effects, in key areas such as amygdala and hippocampus. These lead to, among other things, a capacity reduction of post-stress cognitive processes such as the retrieval of old content. In line with Schwabe et al.^30^, movement selection based on the retrieval of a previously learned rule might be negatively affected by previously experienced stress, and thereby reduce the rule-based efficiency effect.

Similarly, studies addressing the effects of acute social-evaluative stress on motor control demonstrate mixed results with respect to the specific effects on performance^31^. For example, social-evaluative stress has been found to impair athletic performance in golf-putts when the subject tried to retrieve recently acquired explicit golf-knowledge but performance remained stable when mainly implicit knowledge about golf putting was available^32–34^. In relation to speech performance, social-evaluative stress has been associated with an increase in pitch variation when talking^35^. Emotionally stressing stimuli also demonstrated to have an impact on speech and led to prolonged response times in an emotional stroop task in persons who stutter^36^. Studies involving complex motor-control in musical performance showed that social-evaluative stress reduces fine-grained motor-coordination in pianists^37^, but in a different study on social-evaluative stress in musicians it did not amplify motor impairments in pianists with focal dystonia^38^, although stress-induced changes in muscle activation could be observed.

Additional evidence stems from animal studies, where several studies demonstrated that seemingly simple movements like locomotion and grasping can be negatively influenced by acute physical stress^39,40^, i.e. by exposure to forced swimming in cold water, or by fixation. To the best of our knowledge, only Macht et al.^41^ investigated effects of psychological stress on simple goal-directed reaching-grasping movements in humans. They induced emotional stress in patients with Parkinson’s disease and healthy controls by instructing participants to solve mental arithmetic tasks while listening to loud music. Participants had to reach, grasp, and transport a piece of bread, i.e., they repeated the same movement several times. Results indicated that stress did not impair the quality of the movement but rather led to a significantly improved movement-time from the target back to the body in patients and in controls, contrasting the impairing stress-effects on simple movements reported in animal studies^39,40^. Drawing upon the findings by Macht et al.^41^, plan-based movement control in humans might be robust towards or even enhanced by effects of social-evaluative stress. However, the above described heterogeneous picture of stress effects on motor-cognitive performance suggests that the effects of stress on motor-control strongly depend on the task and context of the experimental setting, which requires reevaluating these effects in novel paradigms.

Commonly applied indices for the success of experimental stress manipulations such as the Trier Social Stress Test (TSST) are heart rate (HR), heart rate variability (HRV, e.g., RMSSD) and salivary cortisol^42,43^. Moreover, these indices seem to be tightly linked to motor-cognitive performance. For example, lower HRV values have been identified as predictive for deficiencies in the development of motor-control in premature infants^44^. Eggenberger et al.^45^ found that higher HRV values were associated with cognitive executive dance training in older adults and a recent study by Finke and Schächinger^46^ showed that pharmacologically induced activation of the central sympathetic nervous system (SNS), mimicking the SNS activation observed under conditions of stress, is linked to motor-cognitive performance in response time tasks with varying complexity. The concentration of cortisol also has been linked to motor-cognitive performance: Lautenbach et al.^47^ observed a correlation of increasing salivary cortisol levels with decreasing performance in tennis serves, and Gaysina et al.^48^ observed an association of higher evening cortisol and slower reaction times in older adults who exhibited low cognitive abilities during childhood. Taken together, several studies point towards a strong association of commonly applied stress indices and motor-cognitive performance. We therefore wanted to examine the association of HR, HRV and cortisol with the plan- versus rule-based movement selection performance.

Taken together, several studies have shown differential effects of physiological (e.g., HRV) and endocrine (cortisol) stress markers and acute psychological (or social-evaluative) stress, both in motor execution and on cognitive tasks. We do not know of any studies that have specifically addressed the effects of social-evaluative stress on motor-cognitive plan- versus rule-based motor control. Thus, in the current study we aimed to investigate potentially social-evaluative stress induced changes in plan- and rule-based movement selection. Employing the Trier Social Stress Test (TSST)^42^ in between two sessions of rule- and plan-based motor tasks, we hypothesized that performance in the second session would be affected by stress. The findings regarding effects of social-evaluative stress are heterogenous and seem to strongly depend on the type of the task. Based on the findings by Scheib et al.^7^, who found that the rule-based efficiency advantage increased under conditions of load, we expected the rule-based efficiency effect to be increased due to the stress-manipulation.

To gain an understanding of how physiological and endocrine correlates of social-evaluative stress might change motor performance, we assessed physiological (heart rate (HR) and heart rate variability (HRV)) and endocrine (salivary cortisol) markers. HR and HRV have been associated with different aspects of the autonomic nervous system (ANS), while cortisol reflects activity of the Hypothalamic–Pituitary–Adrenal (hpa) axis. Hence, we exploratorily examined the association of physiological and endocrine markers with behavioral motor-cognitive performance to generate an idea about the possibly underlying mechanisms and energy-systems serving rule- and plan-based movement selection. Additionally, the marker variables allowed us to check whether the stress manipulation was successful, and we expect the control- and the stress-group to exhibit significantly different HR, HRV and cortisol values in response to the social-evaluative stressor.

## Methods

### Participants

Forty-four males without any psychiatric or neurologic disorders, aged 18–32 years (M = 22.95, SD = 3.34), BMI 17.63–28.08 (M = 22.87, SD = 2.58), participated in the study, split randomly into two groups (stress versus control). Eight participants were excluded from the analysis: 5 participants produced more than 20% errors in the movement selection trials, suggesting that they probably did not understand the task, for 1 participant time-measurements were missing due to technical error, 1 participant did not finish the experiment and 1 participant reported to have departed from the instructions to solve the task. Thus, data from 19 stressed and 17 control group participants were included in the analysis. As this was the first study of our group employing stress to study the impact on plan- versus rule-based movement selection and due to limited financial resources, we decided to focus on men initially, avoiding having to account for menstrual cycle effects on stress hormone regulation and requiring larger sample numbers^49^. Sessions took place in the afternoon, between 1 and 6 pm to control for the circadian rhythm of cortisol^50^. Participants indicated their time of wakening, (7 am to 12 pm), and their sleep duration in hours (M = 7.48, SD = 1.03). Each subject had been up at least 2 h before entering the experiment. Group comparisons indicated that stress and control group did not differ regarding their BMI, sleeping time and age (*p* ≥ 0.531). All participants had normal or corrected to normal vision. The study was approved by the Ethics committee of the University of Konstanz. All participants gave written informed consent prior to participation. The study was conducted in accordance with the declaration of Helsinki.

### Material

**Figure 1:**
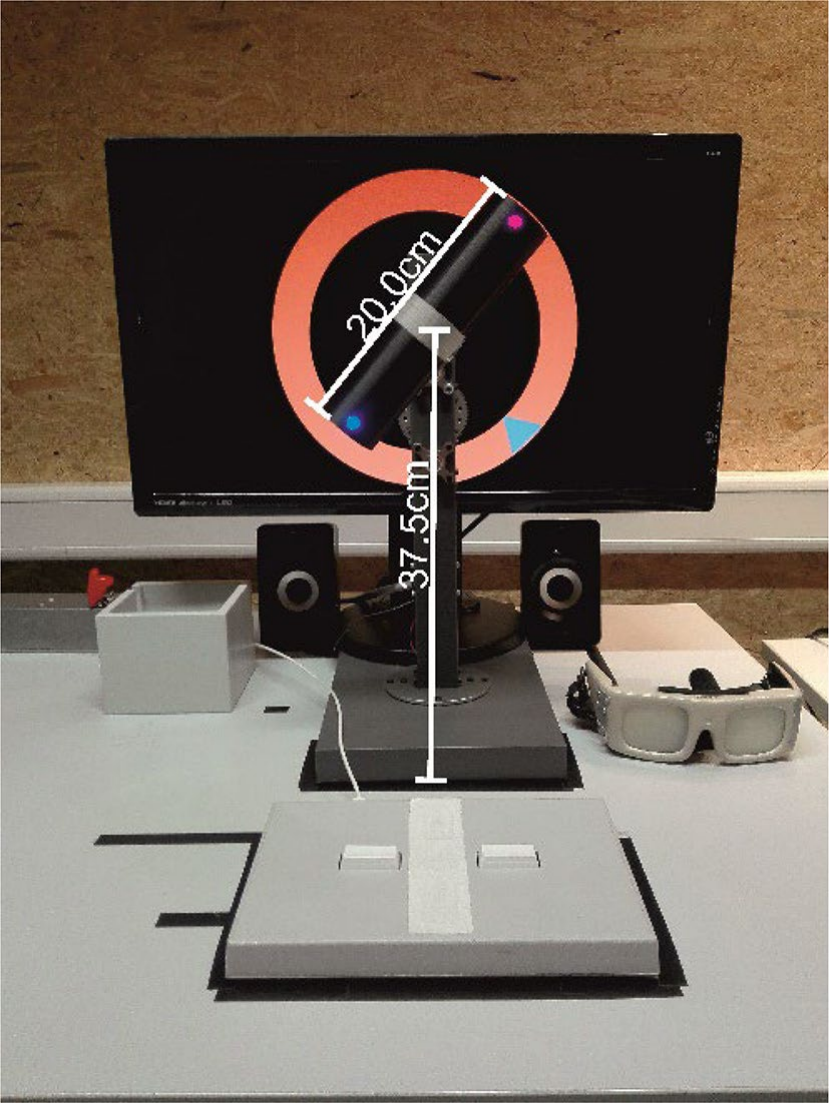
Experimental setup including shutter goggles (shown on the far right), response pad (middle front) and the turning-apparatus in front of a computer screen which was used to display stimulus material (target information): a triangular color cue (here blue, others: pink, yellow, or green) on an orange circle marked the target position. Participants grasped and turned the handle in a way that the same-colored dot on the handle would be aligned with the target arrow (e.g., blue to blue). The handle’s position was at a 90° angle to its target position and its LEDs either shined in the combination yellow-green or pink–blue.

Participants performed the movement selection task with a purpose-built turning-apparatus (Fig. 1). It was composed of a rotatable handle, two light emitting diodes (LEDs) were mounted on it and it was attached to a stand and linked to a small motor that allowed its automatic positioning. For a detailed construction manual see https://github.com/MoCogKonstanz/RPMC. The turning-apparatus was placed in front of a 24-inch computer screen. The location of the handle corresponded to the center of the screen. A response pad (*Lumina RB-540 by Cedrus,* USA) was placed in front of the turning-apparatus. It recorded start and finish of the participant’s movement.

Participants wore shutter goggles (*PLATO Visual Occlusion Spectacles by Translucent Technology Incorporated,* Canada) that switched between opaque (closed) and transparent (open). The goggles obscured the participants’ view while the handle was adjusting itself. Their opening triggered the start of the response-time measurement.

The experimental software *Presentation* (*Neurobehavioralsystems,* USA) was used on a Windows 10 PC. The software triggered the opening and closing of the goggles, positioning of the handle and stimulus presentation. It recorded input from the response pad, i.e., time-measurement. A *NI-DAQ* hardware interface (*Texas Instruments Incorporated,* USA) transmitted information to the turning-apparatus.

Heart rate (HR) was tracked with a *H7 Polar heart rate sensor* (*Polar Electro GmbH Deutschland,* Germany) and the *Heart Rate Variability Logger App* by Altini^51^, which ran on an *iPad* with *iOS 8* (*Apple Inc*., USA).

**Figure 2:**
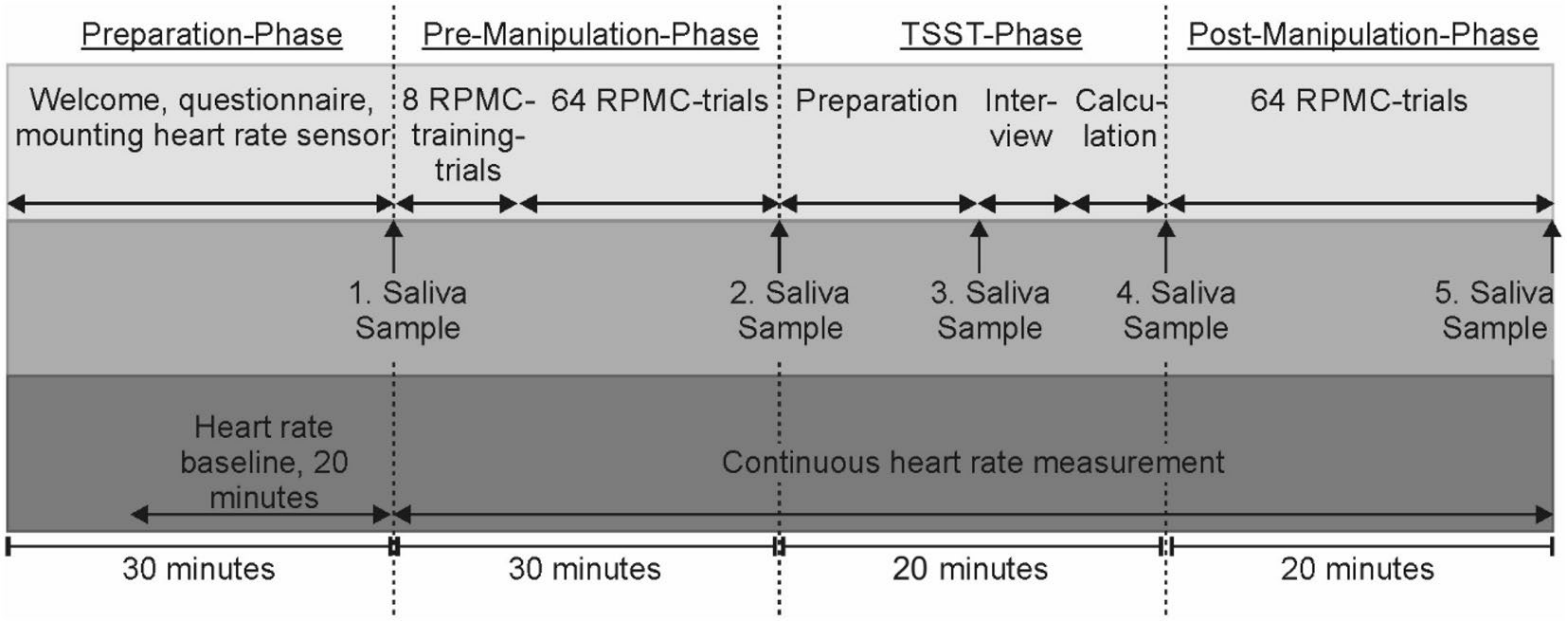
Procedure of the experimental session divided by phases (columns) and features (rows). Top row (pale grey) shows the participant’s tasks throughout the experiment. Middle row (medium grey) displays time points of collection of saliva-samples (cortisol measurement) and the lower row (dark grey) reflects the aggregation of heart rate measures.

Cortisol samples were gathered using *salivettes* (*Sarstedt*, Germany) at − 30, 0, 10, 20 and 40 min in relation to stressor onset (Fig. 2). Participants chewed on the synthetic swab for 1–2 min. After collection, salivettes were stored in a freezer at − 20° Celsius until analysis. Cortisol from saliva was then determined using a time-resolved fluorescence immunoassay with proven reliability and validity^52^.

### Procedure (Fig. 2)

First, participants were instructed to apply the heart rate sensor on their chest. While they filled out a demographic questionnaire and thereafter waited quietly, a 20-min heart rate baseline was recorded.

At the beginning of the pre-manipulation phase, participants provided their first saliva sample. The experimenter instructed the participant on how to do the rule- and plan-based movement selection tasks. Eight training trials and 64 experimental trials followed. Then, the second saliva sample was collected. The TSST stress- or control-manipulation followed. Ten minutes after stressor onset, participants provided the third saliva sample.

After the manipulation, participants provided the fourth saliva sample. Then, participants performed another 64 movement selection trials. After finishing the experiment, the participants gave their final saliva sample and took off the heart rate sensor.

Participants received a debriefing that addressed the focus of the study. Lastly, they received either course credit or a monetary reward (20€).

### RPMC movement selection task

A trial started with the participants placing their right loose fist on the response pad button. The goggles were closed, a tone announced their imminent opening. When the goggles opened, response-time measurement started. Participants released the button of the response pad, thereby ending the response-time and starting the movement-time measurement.

Participants chose between two grips when grasping the handle. They either performed a pronated (palm down) or a supinated (palm up) grip. Participants were provided with two strategies for finding the adequate grip. The color combination of the LEDs indicated the adequate strategy. Color combinations were randomized such that half of the participants solved the pink-blue tasks with the plan-based approach and the green-yellow tasks with the rule-based approach.

For the plan-based approach, participants were instructed to perform a grip that would allow finishing the turning movement in a comfortable position (“I will perform the task as comfortably as possible.”). When participants solved the task according to the rule-based approach, they followed specific instructions when grasping the handle (example-instruction with green and yellow color cues for the rule-based approach: “If the arrow is green, then I will place my thumb on the side with the green light. If the arrow is yellow, then I will place my thumb on the side with the yellow light.”).

After the matching LED was aligned with the color cue, participants pressed the button of the response pad again and the movement-time measurement finished.

The experimenter recorded if the trial was solved correctly. Thus, when participants solved the task with the plan-based approach, finishing the movement in an uncomfortable position was considered as erroneous behavior. When solving the task with the rule-based approach an initial grip that was noncompliant with the rule was recorded as an error.

### TSST-stress-manipulation

#### Active TSST (stress group)

Participants in the stress group were exposed to the TSST. The experimenter informed the participants that they had 10 min to prepare for a job interview. Afterward, participants gave a five-minute mock job speech in front of the TSST-committee (trained confederates, one male and one female member). The committee was instructed to not be compassionate with the participants. They pretended to start a video recording and asked participants to begin with their presentation. Thereafter, participants did an arithmetic task for the next five minutes (counting down in steps of 13 from a random 4-digit number). Each time participants made a mistake, the committee requested them to start over again. Participants received a debriefing after finishing the experiment: the committee revealed that the strict behavior was part of the experimental design and that during the TSST the video setup in fact was not recording.

#### Passive TSST (control group)

Participants in the control group were preparing for a job interview. They had 15 min for the preparation and taking notes, but they were not requested to present, which the participants were made aware of beforehand. Afterward, they did the arithmetic task in written form on a piece of paper that was not evaluated. Participants were informed that there would be no evaluation of their performance.

### Data analysis

Normality of all behavioral as well as physiological data was examined using the Kolmogorov–Smirnov test. For post hoc comparisons, Bonferroni-corrected p-values were reported. Where not stated differently, statistical analyses were conducted with the statistics software SPSS 27^53^.

#### Physiological data

We collected RR intervals of all participants at a sampling rate of 1000 Hz throughout the experiment by means of the Polar H7 sensors. Data was stored in real time on an iPad and then transferred to a desktop PC in the lab running R^54^ and R-Studio^55^ together with the R package *RHRV*^56^. HR data could be immediately extracted using the raw RR intervals after visual inspection of all data and exclusion of erroneous data typically caused by movement artefacts or ectopic beats (less than 3% of continuous data in all subjects). Missing data was then interpolated. For the calculation of heart rate variability (HRV), we chose the root mean square of successive differences (RMSSD), a time-domain based marker of HRV, by employing an in-house R script that allows to define specific intervals. In conjunction with the *RHRV* package, RR intervals were translated to HR, interpolated at a sampling frequency of 4 Hz, and then RMSSD is calculated over each ten-minute interval using 60 s segments with a shift of 30 s. As a final step, to improve data fit for analysis with general linear model procedures, the natural logarithm of the resulting HR and RMSSD values was calculated and entered as dependent variables for statistical analysis. A natural logarithm transformation has been conducted with the cortisol data, too.

**Figure 3:**
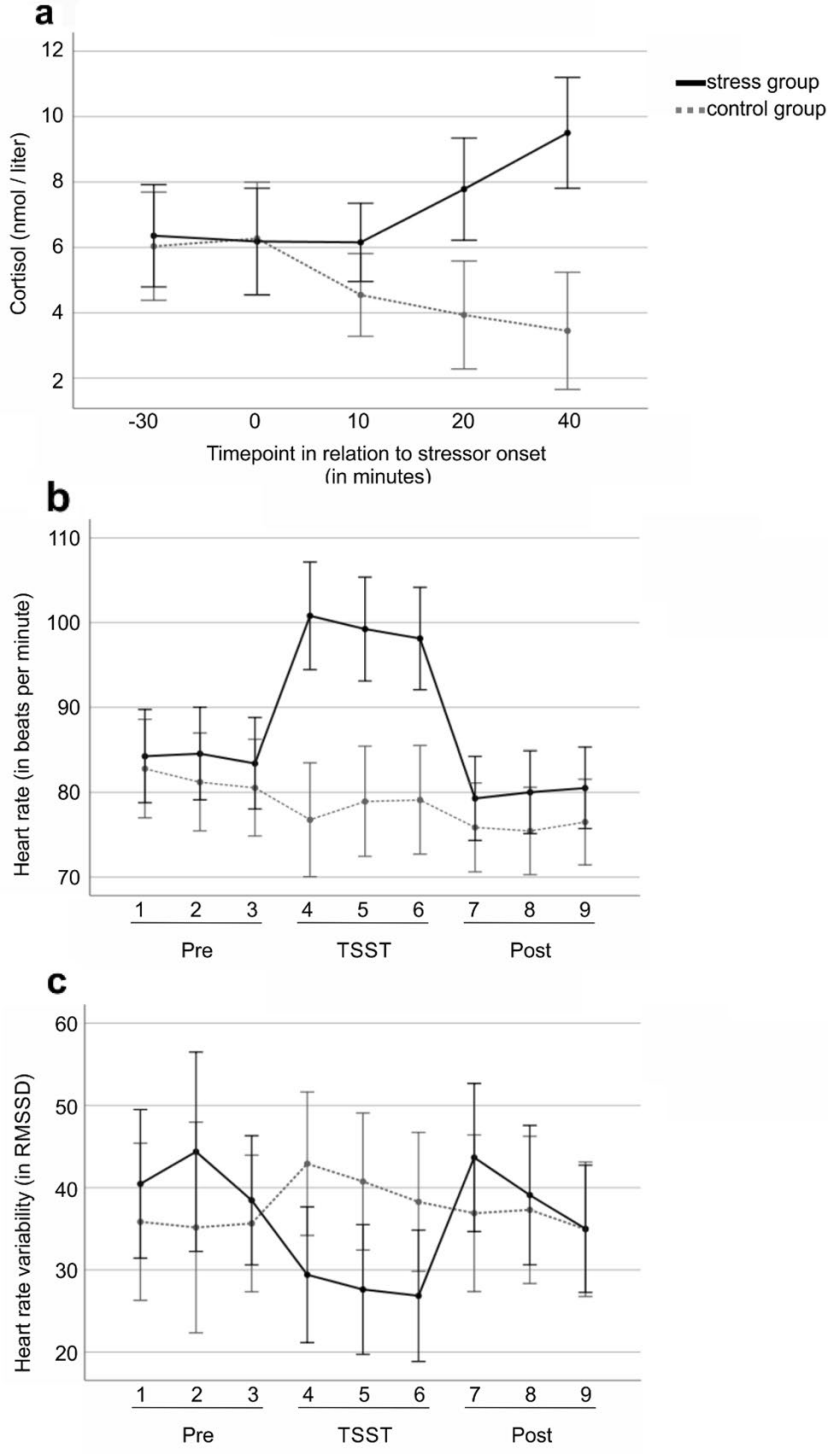
Physiological stress measures. (**a**) Level of salivary cortisol on 5 timepoints throughout the experiment for control and stress group, respectively. Timepoints of measurement were 30 min before stressor onset, at stressor onset, and 10, 20 and 40 min after stressor onset. (**b**) Timeline for HR measurements for control and stress group, respectively. Three sessions (pre-manipulation “pre”, during the manipulation “TSST”, post-manipulation “post”) with three measurements each are shown. (**c**) Time course of HRV measurements in RMSSD throughout the experiment. The RMSSD was calculated in three equally sized time intervals in each of the three sessions “pre”, “TSST”, “post”, resulting in a total of 9 values per participant. Error bars indicate 95% confidence intervals.

Cortisol as well as heart rate data are reported because they reflect stress responses of different physiological systems. HR and HRV reflect a fast, autonomic stress response and cortisol reflects a slow, endocrine stress response^26^. Physiological and endocrine data were compared between groups to prove the effectiveness of the TSST manipulation. The area under the curve with respect to increase (AUC_I_) was calculated for HR, HRV and cortisol concentrations in every participant according to Pruessner et al.^57^. With respect to the different temporal response dynamics, focus was laid on the critical timespan when the stress response came into effect for each system. Thus, we defined the onset of the autonomic stress response to be identical to the onset of the stressor, while the endocrine stress response shows a delay of several minutes after stressor onset^58^. For HR and RMSSD we calculated the AUC_I_ within the manipulation phase (Fig. 3, t3–t7). For salivary cortisol we calculated the AUC_I_ from manipulation onset to the final cortisol measurement (Fig. 3, t2–t5).

Normality could be assumed for all AUC_I_ variables (*p* ≥ 0.086). Between group comparisons were thus performed using t-tests for independent samples.

#### Behavioral data

Technical problems occurred in 1.89% of the trials, which were excluded from analysis. Outlier trials in response and movement-time were detected with the Extreme Studentized Deviate test (ESD) and removed from the data set. The ESD was performed using the Real Statistics Resource Pack Software Release 5.4^59^ for MS Office Excel.

For analyses of behavioral data, we calculated balanced integration scores (BI-Scores), which combined response-time and error rate equally weighted in one score^60^. BI-Scores were calculated for each participant by subtracting the z-standardized mean of correct response times from the z-standardized proportion of correct responses. This strategy allowed controlling for a potential speed-accuracy tradeoff. Higher BI-Scores indicated better performance. Movement-time was analyzed, describing the time needed to reach, grasp, and turn the dowel. All BI-Scores and movement-time variables in stress- and control group were normally distributed (*p* ≥ 0.1).

BI-Scores and movement-time each were analyzed with a 2(group) × 2(session) × 2(approach) mixed design repeated measures ANOVA. Mauchly’s test indicated sphericity of the data.

For follow up analyses, the rule-based efficiency advantage was calculated by subtracting BI-Scores of plan-based from those of rule-based trials. The behavioral change from pre- to post-manipulation RPMC performance was calculated by subtracting pre- from post-manipulation BI-Scores. Analyses of the rule-based efficiency advantage and behavioral change were performed using t-tests for dependent samples.

#### Physiological and behavioral data

The contribution of stress-induced physiological changes in HR, HRV and cortisol to the change in the plan- and rule-based movement selection was examined exploratorily by use of two regression analyses. The dependent variable in each model was the behavioral change. Regression analyses were conducted for the whole sample, employing one regression model for each approach. We included the first four predictors group, cortisol, HR and RMSSD in this sequence. Then, we included the interaction of the factor ‘group’ (coded as dummy variable with 0 = control group, 1 = stress group) together with the variable cortisol. Cortisol was entered early, since it was one of our main predictors and had been found to interact with different cognitive processes in previous studies^20,26,29,61^. As sixth and seventh predictors, we included the interaction of group with the variables HR and HRV (RMSSD), respectively. Both variables have previously been shown to correlate with prefrontal functioning and cognitive performance^62^, hence it is plausible that they might contribute to predicting change in motor-cognitive performance. Participants’ age and BMI were included as additional variables. The assumption of linearity was evaluated by visual inspection of scatterplots of the dependent variable and the predictors, which did indeed suggest a linear relationship. The Durbin–Watson statistic indicated that the assumption of independent residuals was met (1 < Durbin–Watson < 3). Multicollinearity of predictors was evaluated by use of the Variance-Inflation-Factor (VIF), which indicated that multicollinearity between predictors might be present in these models (VIF > 10; tolerance statistics < 0.2). Normality of the residuals was evaluated by use of histograms and PP-plots, which showed that the residuals roughly—although not perfectly—followed a normal distribution.

## Results

### Physiological data

Analysis of the physiological and endocrinological data demonstrated the effectiveness of the stress manipulation.

#### Salivary cortisol

Between group comparison of cortisol levels showed a significantly increased level in the stress group compared to the control group (AUC_I_, t(34) =  − 5.02, *p* < 0.001). The timeline demonstrated strongest effects post manipulation (see Fig. 3a).

#### Heart rate (HR)

Analyses showed that HR was significantly higher in the stress group (AUC_I_, t(34) =  − 6.09, *p* < 0.001). The timeline indicated differences particularly during the TSST manipulation (Fig. 3b).

#### Heart rate variability (RMSSD)

Compared to the control group, RMSSD was significantly reduced in the stress group (AUC_I_, t(25.15) = 2.82, *p* = 0.009). These differences became apparent during the TSST manipulation (Fig. 3c).

### Behavioral data

Behavioral data demonstrated a main effect of approach replicating the rule-based efficiency advantage and a main effect of session indicating a general performance improvement of movement selection and movement-execution when repeating the task. Furthermore, the rule-based efficiency advantage during movement selection was significantly reduced after the manipulation. There was no significant group effect. Results are described in detail below and listed in Table 1.

**Table 1:** Overview of the behavioral results from the movement selection task.

	*df*1	*df*2	*F*	*p*
**BI-Scores**				
Session	1	34	35.63	< 0.001
Approach	1	34	46.77	< 0.001
Group	1	34	0.45	0.508
Session × group	1	34	0.15	0.704
Approach × group	1	34	0.06	0.815
Session × approach	1	34	6.84	0.013
Session × approach × group	1	34	0.86	0.360
**Movement-time**				
Session	1	34	106.63	< 0.001
Approach	1	34	0.87	0.358
Group	1	34	0.14	0.710
Session × group	1	34	0.00	0.961
Approach × group	1	34	2.46	0.126
Session × approach	1	34	1.06	0.312
Session × approach × group	1	34	0.520	0.476

Reported are the results of the repeated measures ANOVA of BI-Scores and movement-time.

#### Movement-time (MT)

Analyses of MT revealed a rather strong main effect of session, F(1,34) = 106.63, *p* < 0.001. MTs in the post-manipulation session were shorter than MTs in the pre-manipulation session.

#### Balanced integration scores (BI-Scores)

We found an overall main effect of session, F(1,34) = 35.63, *p* < 0.001, with higher BI-Scores in the post-manipulation session. Furthermore, we found an overall main effect of approach, F(1,34) = 46.77, *p* < 0.001, indicating significantly higher BI-Scores in movement selection trials solved with the rule-based approach compared to the plan-based approach.

Additionally, we found an interaction of session and approach, F(1,34) = 6.84, *p* = 0.013. Follow-up analyses revealed that the rule-based efficiency advantage (difference between BI-Scores of plan- and rule-based movement selection) was significantly smaller in the post-manipulation session than in the pre-manipulation session (t(35) = 2.68, *p* = 0.011). Trials solved with the plan-based approach improved significantly more from pre- to post-manipulation than trials solved with the rule-based approach.

There was no significant main effect or interaction for the between-subjects factor group (control vs. stress). Due to our a priori hypothesis that social-evaluative stress would increase the rule-based efficiency advantage, we compared this advantage per group. Analyses revealed that to the contrary, a significant reduction of the rule-based efficiency advantage in the stress group could be observed (t(18) = − 2.71, p_adj_. = 0.028). There was no significant difference in this effect for the control group (t(16) = − 1.11, p_adj._ = 0.570). For the mean BI-Scores per group and session, please see Table S1 in the supplementary information.

### Association of physiological measures and behavioral change from pre- to post-manipulation in plan- and rule-based movement selection

To investigate the association of physiological stress markers and plan- and rule-based movement selection, we conducted two regression analyses, i.e., one for the performance change in each approach. The dependent variable per approach was the behavioral change from pre- to post-stress session in plan-based or rule-based movement selection, respectively. The mean performance change in Balanced-Integration-Scores for the plan-based movement selection trials was M = 0.93 (SD = 1.10) in the whole sample, M = 0.97 (SD = 1.17) in the stress- and M = 0.89 (SD = 1.06) in the control-group. For the BI-Score based performance change in the rule-based movement selection trials, a mean of M = 0.44 (SD = 0.57) was computed for the whole sample, with M = 0.32 (SD = 0.60) in the stress- and M = 0.58 (SD = 0.52) in the control group. As predictors, we included group, cortisol, HR, RMSSD and the interaction terms of the dummy-variable ‘group’ with the endocrine (cortisol) and autonomic (HR, RMSSD) stress response. BMI and age were included as control variables. The two resulting models were:

- Plan-based change = b_0_ + b_1_grou* p* + b_2_cortisol + b_3_HR + b_4_RMSSD + b_5_groupXcortisol + b_6_groupXHR + b7groupXRMSSD + b_8_BMI + b_9_Age
  and.
- Rule-based change = b_0_ + b_1_grou* p* + b_2_cortisol + b_3_HR + b_4_RMSSD + b_5_groupXcortisol + b_6_groupXHR + b7groupXRMSSD + b_8_BMI + b_9_Age

**Figure 4:**
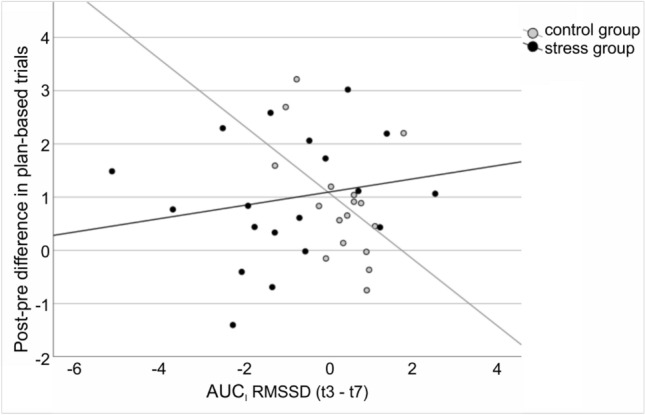
Interaction of AUC_I_ RMSSD (t3–t7) and group (stress versus control) predicting the post–pre difference in plan-based trials. Lines reflect the linear regression lines per group. Grey dots represent participants from the control group and black dots represent participants from the stress group.

Results are summarized in Table 2. The model for the performance change in plan-based trials was significant (F(9,26) = 3.105, *p* = 0.011) and accounted for 51.8% of variance. The predictors HR and RMSSD were both significant, as well as the interaction of group and RMSSD, which is displayed in Fig. 4. In contrast, the model predicting the performance change in rule-based trials was not significant (F(9,26) = 1.336, *p* = 0.267). The model explains 31.6% of variance. None of the predictors in this model were significant.

**Table 2:** Outcome from the regression analyses.

DV	Model evaluation			Regression coefficients				
	F	*p*	R^2^	Predictor variable	b	SE	t	*p*
Plan-based performance improvement	3.11	0.011	0.518	(Intercept)	4.805	1.683	2.855	0.008
				Group	1.125	0.581	1.938	0.064
				AUCI cortisol	0.001	0.026	0.048	0.962
				AUCI HR	− 2.722	1.273	− 2.139	0.042
				AUCI RMSSD	− 1.124	0.369	− 3.049	0.005
				BMI	− 0.134	0.072	− 1.867	0.073
				Age	− 0.036	0.056	− 0.640	0.528
				Group × AUCI cortisol	− 0.040	0.031	− 1.300	0.205
				Group × AUCI HR	1.117	1.462	0.764	0.451
				Group × AUCI RMSSD	1.147	0.397	2.892	0.008
Rule-based performance improvement	1.34	0.267	0.316	(Intercept)	0.174	1.034	0.169	0.867
				Group	0.018	0.357	0.050	0.961
				AUCI cortisol	− 0.007	0.016	− 0.465	0.646
				AUCI HR	0.010	0.782	0.013	0.989
				AUCI RMSSD	− 0.288	0.227	− 1.271	0.215
				BMI	− 0.041	0.044	− 0.926	0.363
				Age	0.059	0.034	1.711	0.099
				Group × AUCI cortisol	0.009	0.019	0.468	0.644
				Group × AUCI HR	− 0.808	0.898	− 0.900	0.376
				Group × AUCI RMSSD	0.221	0.244	0.909	0.372

Displayed is the outcome of the ANOVA that evaluates whether the current model predicts the dependent variable better than assuming the mean of the dependent variable. R^2^ indicates the share of variance in the dependent variable explained by the model. Further, the regression coefficients with their corresponding t- and *p*-values are displayed for each predictor contributing to the current model.

## Discussion

In the current study, we examined the effects of psychosocial stress on rule- versus plan-based movement selection. Subjects performed the movement selection experiment twice, once before and once after the stress or control manipulation, respectively.

Based on prior findings indicating that increased workload induced stronger rule-based efficiency effects we suspected an increased rule-based efficiency effect after stress. However, this was not confirmed. Instead, we observed a general rule-based efficiency advantage. Rule-based performance as indicated via BI-scores, a combination of response-time and error rates, was superior to plan-based performance. We also observed a main effect of session, where we saw a general performance improvement of movement selection and movement-execution over time. Furthermore, the rule-based efficiency advantage during movement selection was significantly reduced in the second session, regardless of the condition subjects were in. There was no significant group effect, suggesting that being in the stress or control condition did not influence this improvement per se. However, to examine our a priori hypothesis that stress would increase the rule-based efficiency advantage, we tested whether the advantage would change from pre- to post-session per group. Results showed that the reduction of the rule-based efficiency advantage was significant in the stress group only, which might have driven the overall reduction observed for the whole sample that is seemingly due to a larger improvement in plan-based than in rule-based tasks. This, if anything, was opposite to our initial hypothesis. It seems to suggest that a training effect regardless of stress or control condition as well as a potentially stress-induced moderate activation—possibly similar to what was observed by Yerkes and Dodson^19^—allowed subjects to improve their plan-based performance the second time around, while the rule-based performance did not improve performance to the same extent.

Further, we measured heart rate and cortisol levels throughout the task. From the analysis of heart rate change, cortisol change, and heart rate variability dynamics, we derived markers of the hypothalamic pituitary adrenal (hpa) axis (via cortisol) and of the autonomic nervous system (ANS, via HRV and HR). Thus, we were able to generate an impression of the association of each of these systems with movement selection performance under stress and control. A regression model predicting performance change in plan-based movement selection from the factor group (stress versus control), its interaction with stress-induced changes in cortisol, HR and RMSSD and the control variables BMI and age was significant and explained 51.8% of variance. The predictors HR, RMSSD as well as the interaction of group with RMSSD contributed significantly to the model. This finding points towards the modulation capability of the plan-based approach to movement selection that seems to be primarily driven by changes in the ANS, reflected by changes in HR and RMSSD in the current study. Especially RMSSD seems to reflect effects of psychosocial stress on plan-based movement selection, as indicated by the significant interaction of group and RMSSD. RMSSD is a measure for HRV, which is a correlate for parasympathetic nervous system (PNS) activation and vagal tone^63^. As can be seen in Fig. 4, the plan-based movement performance of participants in the stress group, where HRV is generally reduced, seems to benefit when RMSSD is rather unaffected and is not reduced by much. In the control group, where HRV seems to slightly increase, plan-based movement selection improves the most when RMSSD does not strongly increase. Taken together, plan-based movement-selection seems to improve the most when RMSSD—and probably also parasympathetic activation—is rather constant and does neither increase nor decrease. One may consider the PNS as a system that is most active during periods of rest^64^, but also during relaxing tasks^65^, tasks that are considered easy, or boring^66^ and sustained attention while engaging in a pleasant task^67^. Hence, it could be argued that subjects in the control group were improving their performance less the second time around the more they experienced the task as easy, but also as potentially boring. In the stress group, however, it could be argued that the withdrawal of parasympathetic activation might lead to adverse circumstances for the training of plan-based movement selection. Besides RMSSD, also HR seems to be of importance when it comes to predicting plan-based movement selection enhancement. However, the interpretation of what causes change in HR is not straightforward. HR is a measure that is influenced by both branches of the ANS, the sympathetic as well as the parasympathetic^68^. This means, on the one hand, that the increase of HR might be an effect of sympathetic activation, but on the other hand, a moderate increase of HR could also reflect parasympathetic withdrawal. This latter argument seems to be in line with the RMSSD x group interaction, which suggests for the stress group that decrease of RMSSD (i.e., parasympathetic withdrawal) appears to be associated with smaller plan-based movement selection enhancement. All in all, the current findings suggest that the autonomic nervous system activity is predictive for plan-based movement selection improvement. Especially the HRV, as reflected by RMSSD, seems to be a strong modulator, particularly for the control group that shows weaker performance improvement the more the RMSSD increases.

When it comes to the prediction of the rule-based movement selection performance increase, ANS activity seemingly does not play a role that is as important as for the plan-based approach. In fact, our model predicting rule-based performance increase was not significant, explaining 31.6% of variance. None of the predictors seemed to be of importance for the model. In other words, none of the stress markers could predict rule-based performance increase, which itself was smaller than the plan-based performance enhancement. This finding underpins the result that rule-based movement selection performance is rather stable and unsusceptible to external influences.

Although it should be kept in mind that these analyses were exploratory and interpretation is limited due to multicollinearity, they seem to indicate the finding that the plan-based approach to movement-selection is susceptible to modulation by external influences, while the rule-based approach seems to be rather invariant. This is especially interesting regarding the fact that the visually observable movement is always the same, independent from the applied tactic. In other words, the approach is decisive when it comes to the modulating role of repetition or other external factors like psychosocial stress.

Comparable results have been reported earlier in the literature, although plan- versus rule-based movement control has—to the best of our knowledge—not yet been directly compared in one task. For example, Lautenbach et al.^47^ examined the relationship of salivary cortisol concentration and motor performance in an athletic task. In their study, participants were asked to perform second tennis-serves prior to and after conducting a stressful arithmetic task. The authors found that stress induced salivary cortisol explained about 19% of variance in the performance drop of second tennis-serves after the stressful intervention. Hence, planning and conducting second tennis-serves as an example of a complex motor task seems to be modulated by psychosocial stress. In contrast, motor-cognitive performance in a go-no-go task seemed to be rather invariant towards effects of psychosocial stress, but only as long as participants used if–then-rules to perform the task^69^.

In the current study several known effects have been replicated, supporting the reliability of the implemented tasks. First, we replicated the rule-based efficiency effect that has been demonstrated across several movement selection studies using a similar design in healthy young adults^4–7^. The main effect of approach indicated better BI-Score performance in trials solved with the rule-based approach compared to trials solved with the plan-based approach. Second, the overall main effect of session showed better performance in the post-manipulation session compared to the pre-manipulative session in either group. Both, the movement selection as described by BI-Scores as well as the movement-execution described by movement-time improved over time. These results reflect long known training effects in response- and movement-time measures due to repeated exposure to experimental procedures^70,71^. We additionally found that the rule-efficiency effect was significantly reduced in the post-manipulation session. Specifically, performance in plan-based tasks improved significantly more than the performance in rule-based tasks. Evidence accumulates across studies^5,6^ that rule-based action selection is pretty efficient from the start and that plan-based action selection has more potential for improvement due to repetition. Third, we were able to demonstrate that the stress manipulation using the TSST was effective. This has been demonstrated by between group effects on the physiological markers heart rate, heart rate variability and concentration of salivary cortisol. Similar physiological effects of the TSST have been reported in several studies that employed this specific stress manipulation^20,49,72^.

There are several limitations that need to be kept in mind when considering the results of the current study. We tested only men; thus, we can’t say whether results generalize to women, and whether menstrual cycle phase effects might be present. Methodological limitations of the current study include a limited time span of cortisol measures—subjects were not past their peak at the last cortisol measure and thus possible group differences in the recovery period might have gone undetected. Therefore, it might be possible that the cortisol-dependent stress effects were not completely developed during the post-manipulation session. HR was applied as indicative of autonomic nervous system activation encompassing both branches (both sympathetic and parasympathetic) in the current study. However, future studies would benefit from applying pure sympathetic markers such as pre-ejection period or skin conductance in addition to heart-rate variability. Finally, for the interpretation of the regression analyses, one should keep in mind that they were exploratory, and that multicollinearity of the predictors limits their interpretational value. Hence, these analyses should rather be the base for generating novel hypotheses on the interaction of ANS activity with motor cognitive tasks. Regarding the RPMC-experiment, the movement selection task was very simple. Future studies should implement plan- and rule-based movement selection tasks and interactions with more complex every-day objects, also the evaluation of the movement itself may provide additional information about underlying mechanisms. Gallivan et al.^73^ argue that movements towards objects are under online control and visual feedback allows to constantly update the movement. Future studies may therefore profit from additionally implementing kinematic analyses of the movements to contribute to a more comprehensive understanding of how stress affects movement selection as well as execution processes involved in motor-cognitive tasks.

Taken together, the current findings suggest that stress and its effects might play a considerable role in motor-cognitive performance. They indicate that rule-based movement selection might be rather resistant towards psychosocial stress-induced changes in hpa and ANS parameters, whereas plan-based movement selection might be prone to ANS effects, especially to variations in RMSSD. This modulating effect might be of importance in both directions: parasympathetic withdrawal (reflected by *decrease* in RMSSD) as well as parasympathetic activity increase (reflected by *increase* in RMSSD) appear to be detrimental for plan-based movement selection performance enhancement. Instead, a steady state of parasympathetic activity might be beneficial. To our knowledge, the current study was the first to examine the effects of psychosocial stress on rule- versus plan-based approaches to movement selection. Hence the findings reported in the current piece of work need replication in a larger and more diverse sample and should be tested in more complex motor-cognitive tasks.

## Supplementary Information

Supplementary Information.
