# Supplementary material for: Selective effects of psychosocial stress on plan based movement selection
Source: Sci Rep. 2022 Mar 30;12:5401. doi: 10.1038/s41598-022-09360-0 (PMC8967871; doi:10.1038/s41598-022-09360-0)
Supplement: Supplementary file 1 — Supplementary Information. [file 41598_2022_9360_MOESM1_ESM.pdf]

Selective effects of psychosocial stress on plan based movement selection

Sarah E. M. Stoll[1,2], Leonie Mack[1], Jean P. P. Scheib[1,2], Jens Pruessner[1]

& Jennifer Randerath[1,2*]

[1] Department of Psychology, University of Konstanz, 78464 Konstanz, Germany
[2] Lurija Institute for Rehabilitation and Health Sciences at the University of Konstanz, Schmieder Foundation for Sciences and Research, 78476 Allensbach, Germany
* Corresponding author

Supplementary Information

Group	Variable	Mean	SD	MD	Min.	Max.
Control Group N = 17	BI-Score plan-based tasks pre	-1.01	1.40	-0.78	-3.84	1.14
	BI-Score rule-based tasks pre	0.07	1.01	0.40	-2.57	1.26
	BI-Score plan-based tasks post	-0.12	1.20	0.40	-2.96	1.28
	BI-Score rule-based tasks post	0.65	0.86	0.88	-1.35	1.72
	MT plan-based tasks pre (in ms)	2396.84	460.62	2409.37	1652.58	3590.54
	MT rule-based tasks pre (in ms)	2425.83	530.18	2303.15	1651.40	3935.17
	MT plan-based tasks post (in ms)	2170.43	424.16	2151.14	1595.21	3384.44
	MT rule-based tasks post (in ms)	2169.86	465.44	2152.00	1597.43	3558.16
	Salivary cortisol at t1 (in nmol/liter)	6.04	3.40	5.07	0.97	14.13
	Salivary cortisol at t2 (in nmol/liter)	6.27	4.13	5.53	0.97	19.23
	Salivary cortisol at t3 (in nmol/liter)	4.55	1.75	4.23	0.87	7.63
	Salivary cortisol at t4 (in nmol/liter)	3.93	1.85	3.64	1.06	7.55
	Salivary cortisol at t5 (in nmol/liter)	3.45	1.46	3.19	0.66	6.38
	HR pre (in bpm)	81.51	9.29	82.04	68.06	96.49
	HR TSST (in bpm)	78.27	9.84	76.66	63.28	96.45
	HR post (in bpm)	75.94	8.52	75.55	61.08	91.91
	HRV pre (in rMSSD)	35.55	13.61	32.67	18.20	61.52
	HRV TSST (in rMSSD)	40.64	17.92	37.64	11.82	81.60
	HRV post (in rMSSD)	36.38	12.36	33.45	20.56	61.11
Stress Group N = 19	BI-Score plan-based tasks pre	-0.89	1.30	-0.76	-3.70	1.00
	BI-Score rule-based tasks pre	0.43	0.91	0.56	-1.95	1.68
	BI-Score plan-based tasks post	0.08	1.12	0.39	-2.27	1.55
	BI-Score rule-based tasks post	0.75	0.56	0.81	-0.99	1.37
	MT plan-based tasks pre (in ms)	2497.98	539.36	2441.57	1444.62	3382.60
	MT rule-based tasks pre (in ms)	2444.64	466.62	2508.23	1501.31	3213.44
	MT plan-based tasks post (in ms)	2257.09	495.27	2190.97	1269.64	2995.56
	MT rule-based tasks post (in ms)	2198.58	451.53	2206.86	1279.70	2925.81
	Salivary cortisol at t1 (in nmol/liter)	6.36	3.32	5.89	1.39	13.75
	Salivary cortisol at t2 (in nmol/liter)	6.18	2.80	7.11	2.17	10.89
	Salivary cortisol at t3 (in nmol/liter)	6.15	3.12	5.89	2.13	15.36
	Salivary cortisol at t4 (in nmol/liter)	7.78	4.26	7.53	0.93	15.50
	Salivary cortisol at t5 (in nmol/liter)	9.50	4.80	8.99	1.38	19.80
	HR pre (in bpm)	84.09	13.22	86.85	59.47	108.65
	HR TSST (in bpm)	99.39	15.14	100.26	73.02	123.87
	HR post (in bpm)	79.94	11.79	82.19	53.68	102.21
	HRV pre (in rMSSD)	41.10	22.13	34.23	15.94	87.68
	HRV TSST (in rMSSD)	27.96	14.61	25.88	8.45	68.97
	HRV post (in rMSSD)	39.25	20.77	33.93	14.24	103.56

Supplementary Table S1. Mean, standard deviation (SD), minimum (Min.) and maximum (Max.) values of behavioral variables in Balanced Integration Scores (BI-Score) and movement-time (MT) and of the physiological stress measures salivary cortisol, heart rate (HR) and heart rate variability (HRV).

Abbreviation	Meaning
BI-Score	balanced integration score
hpa axis	hypothalamic pituitary adrenal axis
HR	heart rate
HRV	heart rate variability
MT	movement time
PNS	parasympathetic nervous system
RMSSD	root mean square of successive differences (HRV measure)
RPMC paradigm	rule-plan-motor-cognition paradigm
SNS	sympathetic nervous system
TSST	Trier Social Stress Test

Supplementary Table S2. Table of abbreviations.
